# Intersigmoid Hernia Masquerading as Small Bowel Stricture: A Diagnostic Challenge

**DOI:** 10.7759/cureus.103370

**Published:** 2026-02-10

**Authors:** Mina Fouad, Boluwatife Ayantunde, Johnathan Evans

**Affiliations:** 1 General Surgery, Nottingham University Hospitals, Nottingham, GBR; 2 Surgery, Nottingham University Hospitals, Nottingham, GBR

**Keywords:** internal hernia, intersigmoid fossa, laparotomy, sigmoid mesocolon, small bowel obstruction

## Abstract

Internal hernias are an exceptionally uncommon cause of small bowel obstruction. Among these, sigmoid-mesocolon hernias, particularly intersigmoid hernias, are one of the rarest subtypes.

A 66-year-old male with no history of abdominal or groin surgery presented with a six-hour history of acute lower abdominal pain and 48 hours of absolute constipation. Computed tomography demonstrated dilated small bowel loops with a transition point in the left iliac fossa. Initial conservative management failed to relieve symptoms. Midline laparotomy revealed a viable loop of mid-ileum herniated into a peritoneal pouch adjacent to the sigmoid colon, consistent with an intersigmoid internal hernia. The bowel was reduced, and the peritoneal recess was obliterated. The postoperative course was uneventful, and the patient was discharged home on day four.

Intersigmoid internal hernia is an exceedingly rare cause of small bowel obstruction and should be considered in patients without prior abdominal surgery. Early surgical intervention remains key to preventing strangulation and ensuring favourable outcomes.

## Introduction

Small bowel obstruction is a common surgical emergency, accounting for up to 15-20% of hospital admissions for acute abdomen [1]. While the predominant causes include postoperative adhesions, external hernias, and malignancy, internal hernias are an uncommon aetiology, responsible for only 0.6-5.8% of cases [2,3].

Internal hernias occur when a viscus protrudes through a congenital or acquired peritoneal or mesenteric aperture within the abdominal cavity. Among the recognised subtypes, sigmoid-mesocolon-related hernias (intersigmoid, transmesosigmoid, and intramesosigmoid) are particularly rare [4].

An intersigmoid hernia arises when small bowel loops herniate into the congenital intersigmoid fossa, a peritoneal recess located at the lateral attachment of the sigmoid mesocolon. Although typically asymptomatic, bowel entrapment within this pouch can cause acute obstruction, posing a diagnostic challenge due to its non-specific clinical and radiological features [5].

We present a rare case of small bowel obstruction secondary to an intersigmoid internal hernia in a 66-year-old male, successfully managed with prompt surgical intervention. This report highlights the diagnostic and operative challenges associated with sigmoid-related internal hernias and highlights the importance of considering congenital peritoneal recesses in patients presenting with obstruction without a history of prior abdominal surgery.

## Case presentation

A 66-year-old man presented to the emergency department with a six-hour history of sudden-onset, sharp lower abdominal pain. He had not passed a bowel motion or any flatus for the preceding two days. He reported no previous similar episodes, vomiting, or overt abdominal distension. He had no history of abdominal or groin surgery. On examination, he was haemodynamically stable, with localised tenderness in the left lower quadrant. Laboratory investigations were unremarkable, including a normal serum lactate of 1.3 mmol/L.

**Figure 1:**
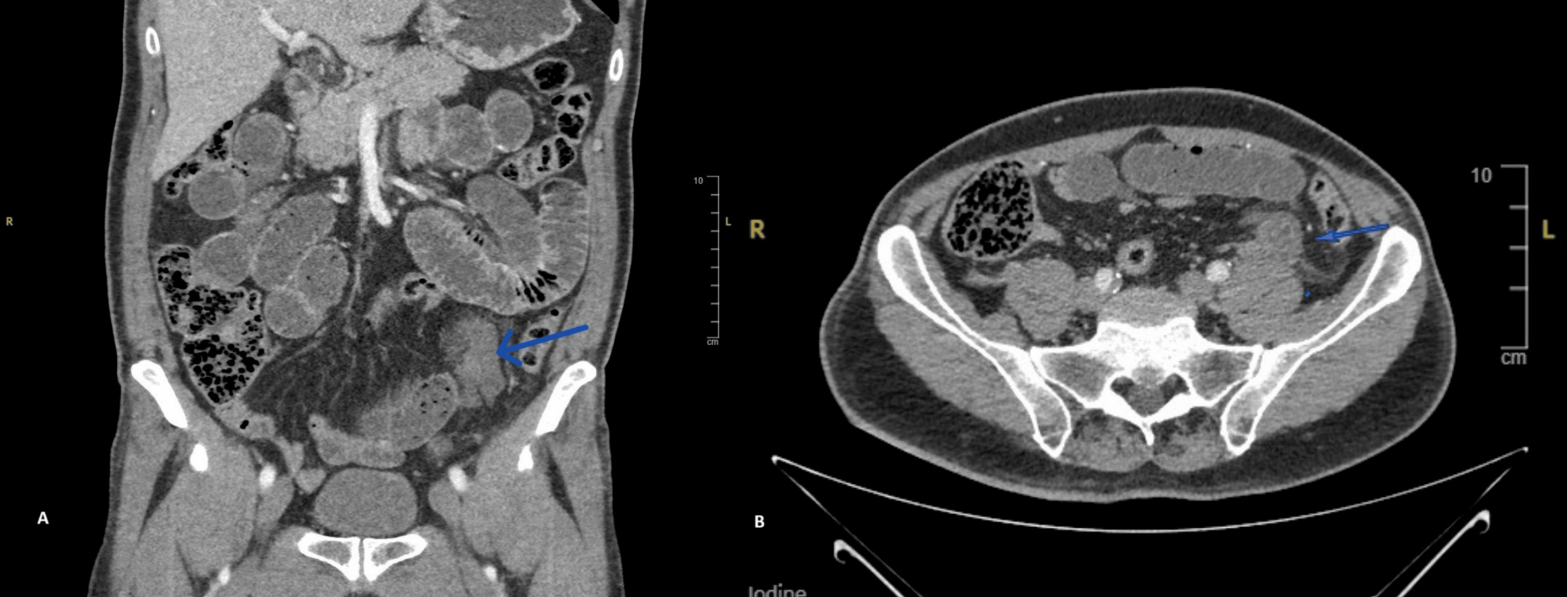
Computed tomography (CT) scans. (A) Coronal view: The image shows the overall abdominal anatomy with multiple dilated small bowel loops, suggestive of obstruction. (B) Axial view – low pelvis: The image demonstrates clustered small bowel loops and a transition point in the left iliac fossa (indicated by the blue arrow), adjacent to the sigmoid colon. This corresponds to the location of the intersigmoid internal hernia.

Contrast-enhanced computed tomography of the abdomen and pelvis demonstrated dilated proximal small bowel loops with a distinct transition point in the left iliac fossa. At this level, a short segment of mid-ileum exhibited mural oedema and mesenteric stranding. The radiological impression was small bowel obstruction, possibly secondary to an inflammatory or neoplastic stricture in the mid-ileum, highlighting the non-specific nature of the findings. In retrospect, the clustered small bowel loops and the transition point adjacent to the sigmoid colon, though initially misinterpreted, were subtle but classic signs of a sigmoid-related hernia (Figure 1).

**Figure 2:**
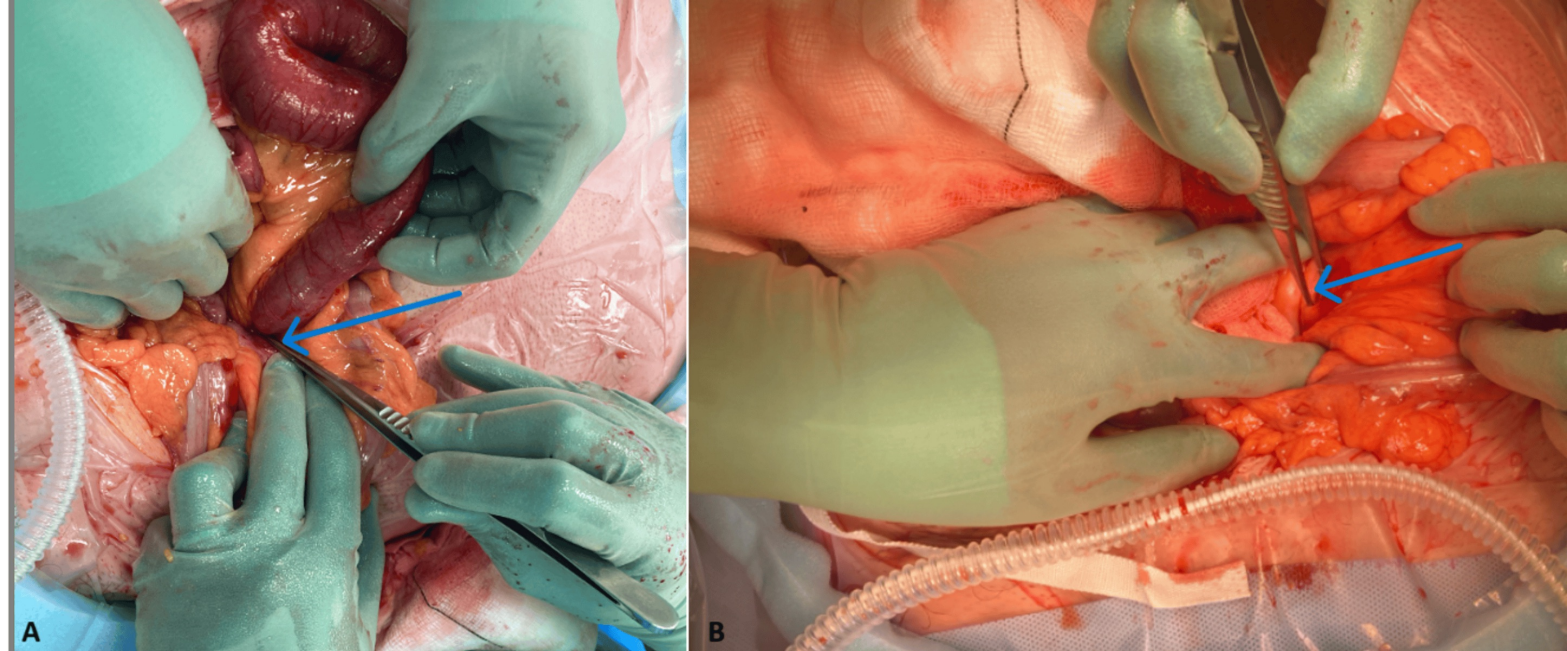
Intraoperative findings during laparotomy. (A) The image shows the loop of mid-ileum (blue arrow) that had herniated into the intersigmoid fossa and was successfully reduced through the peritoneal opening. (B) The image demonstrates the peritoneal recess adjacent to the sigmoid colon, confirming the intersigmoid internal hernia (blue arrow).

As there was no clinical improvement after 24 hours of conservative management (nasogastric decompression and intravenous fluids), a lower midline laparotomy was performed due to diagnostic uncertainty and the surgeon's choice. Intraoperatively, a loop of mid-ileum was found to have herniated into a peritoneal recess (the intersigmoid fossa) adjacent to the sigmoid colon, confirming an intersigmoid internal hernia. The peritoneal pouch measured approximately 6 cm in depth, had a tight neck, and did not involve a full-thickness mesenteric defect. The herniated bowel was viable and was reduced without difficulty (Figure 2).

The congenital intersigmoid fossa was subsequently obliterated with sutures to prevent recurrence. The patient resumed oral intake on postoperative day two and was discharged home on day four in good condition.

## Discussion

Internal hernias are an uncommon cause of small bowel obstruction, accounting for approximately 0.6-5.8% of all cases. The present case highlights the particular rarity of sigmoid-mesocolon hernias, which are classified into intersigmoid, intramesosigmoid, and transmesosigmoid types, depending on the anatomical nature of the defect [1-3].

An intersigmoid hernia occurs when small bowel loops herniate into the intersigmoid fossa, a congenital peritoneal recess formed by the attachment of the sigmoid mesocolon to the posterior abdominal wall during embryological development. In contrast, intramesosigmoid hernias involve a defect in one leaf of the sigmoid mesocolon, while transmesosigmoid hernias traverse a full-thickness mesocolic defect [4]. The present case is consistent with an intersigmoid hernia, in which a loop of viable small bowel was entrapped within a peritoneal pouch rather than a true mesenteric defect.

Preoperative diagnosis of sigmoid-related internal hernias is challenging. The clinical presentation is non-specific and often indistinguishable from adhesive obstruction. Computed tomography remains the investigation of choice and may demonstrate clustered small bowel loops in the left lower quadrant, converging mesenteric vessels, and a transition point adjacent to the sigmoid colon [5,6]. However, CT findings are frequently ambiguous, lacking the specific features required to distinguish internal herniation from other causes of obstruction. Consequently, radiologists often attribute the findings to more prevalent aetiologies, favouring diagnoses such as adhesions or intrinsic bowel pathology. This diagnostic bias frequently leads to these hernias being misidentified as inflammatory or neoplastic strictures, obscuring the true anatomical defect. This diagnostic pitfall was clearly illustrated in the present case.

Early surgical intervention is essential, as delayed recognition may result in bowel strangulation and necrosis. Operative exploration provides both diagnostic clarity and therapeutic benefit, allowing direct visualisation, reduction of herniated loops, and definitive closure of the defect [7]. In the current case, reduction and obliteration of the peritoneal recess achieved complete resolution without the need for bowel resection.

In summary, an intersigmoid internal hernia is a rare but important cause of small bowel obstruction, particularly in patients with no prior abdominal surgery. A high index of suspicion, combined with timely imaging and a low threshold for operative exploration, is key to avoiding morbidity.

## Conclusions

Intersigmoid internal hernia is a rare but clinically significant cause of small bowel obstruction, particularly in patients without a history of abdominal surgery. Its non-specific presentation and subtle radiological features make preoperative diagnosis challenging. Awareness of this entity and early operative intervention are essential to prevent strangulation and ensure favourable outcomes.
